# Noninvasive prescreening of pediatric adenoid hypertrophy using quantified MFCC statistics and clinical features: development and external validation

**DOI:** 10.3389/fbioe.2026.1740863

**Published:** 2026-02-05

**Authors:** Yirun Jiang, Xiaoyu Wang, Wen Hu, Shizhen Zou, Lili Peng, Zhan Wang, Siyuan Hou, Jinrang Li, Jun Tai

**Affiliations:** 1 Capital Center for Children’s Health, Capital Medical University, Capital Institute of Pediatrics, Beijing, China; 2 Capital Institute of Pediatrics, Chinese Academy of Medical Sciences and Peking Union Medical College, Beijing, China; 3 College of Otolaryngology Head and Neck Surgery, The 6th Medical Center, National Clinical Research Center for Otolaryngologic Diseases, Chinese PLA General Hospital, Beijing, China

**Keywords:** adenoid hypertrophy, decision-curve analysis, external validation, locked cutoff, machine learning, MFCC, patient-level validation, pediatric OSA

## Abstract

**Background:**

Adenoid hypertrophy (AH) is a leading cause of pediatric obstructive sleep apnea, yet first-line diagnostics are invasive or resource-intensive. We developed and externally tested a low-cost, noninvasive screening model that fuses quantitative voice features with routine clinical variables for pre-endoscopy and primary-care triage.

**Methods:**

In a dual-center cross-sectional study (N = 202), Center 1 (Capital Center for Children’s Health, Capital Medical University, n = 161) served as the development cohort and Center 2 (College of Otolaryngology Head and Neck Surgery, The 6th Medical Center, National Clinical Research Center for Otolaryngologic Diseases, Chinese PLA General Hospital, Beijing, China, n = 41) as the independent external cohort. Children produced sustained/a/phonations; Mel-frequency cepstral coefficients (MFCCs) were summarized into fixed statistics and combined with readily available clinical information. Modeling used patient-level aggregation with stratified 10-fold cross-validation in development. The final classifier was selected by a joint criterion of AUC and average precision (AP), then a single Youden-derived locked cutoff was determined in the development set and applied unchanged to the external cohort. Discrimination (AUC/AP), calibration (Brier score, slope, intercept), and clinical utility were evaluated.

**Results:**

Internal performance was stable (AUC = 0.81; AP = 0.84). On the small external cohort, discrimination remained (AUC = 0.79; AP = 0.88). At the locked cutoff, the model achieved clinically actionable sensitivity/specificity with balanced F1. Calibration was acceptable (Brier = 0.20, slope = 0.71, intercept = 0.94). Decision-curve analysis showed positive net benefit across a wide range of threshold probabilities versus “treat-all” and “treat-none.” SHAP explainability indicated MFCC variability-related features and a subset of airway-symptom clinical variables as leading contributors, aligning with hyponasal resonance changes in AH.

**Conclusion:**

A patient-level model with a locked decision threshold showed preservation of discrimination in a small external cohort, supporting a practical pathway for noninvasive, low-overhead AH triage prior to nasoendoscopy. Prospective multicenter studies are warranted.

## 1 Introduction

Adenoid hypertrophy (AH) is the most common anatomic cause of pediatric obstructive sleep apnea (OSA) in preschool- and school-aged children (Wang et al., 2019). Persistent AH can lead to sleep architecture disruption, impairments in cognition and attention, craniofacial growth abnormalities, and other adverse outcomes (Fagundes et al., 2022; Menzies et al., 2022; Don et al., 2024). Given the high disease burden yet reversibility, adenoidectomy (with or without tonsillectomy) remains among the most frequently performed pediatric surgeries worldwide; early identification and risk stratification therefore carry clear public-health and clinical value (McDermott and Liang, 2019).

Current first-line evaluations rely primarily on two modalities: lateral nasopharyngeal radiography and flexible nasopharyngoscopy. The former assesses structural obstruction using ratios such as the adenoid–nasopharynx (A/N) ratio; the latter grades the proportion of choanal obstruction caused by the adenoids (Fujioka et al., 1979; Cassano et al., 2003; Parikh et al., 2006). However, radiography entails ionizing radiation and requires equipment and standardization, whereas nasopharyngoscopy—despite direct visualization—is invasive, demands substantial cooperation from children, and is costly to deploy at scale in primary-care or school screening settings (Baldassari and Choi, 2014). These practical constraints motivate the development of safe, low-barrier, and reproducible noninvasive screening technologies.

**FIGURE 1:**
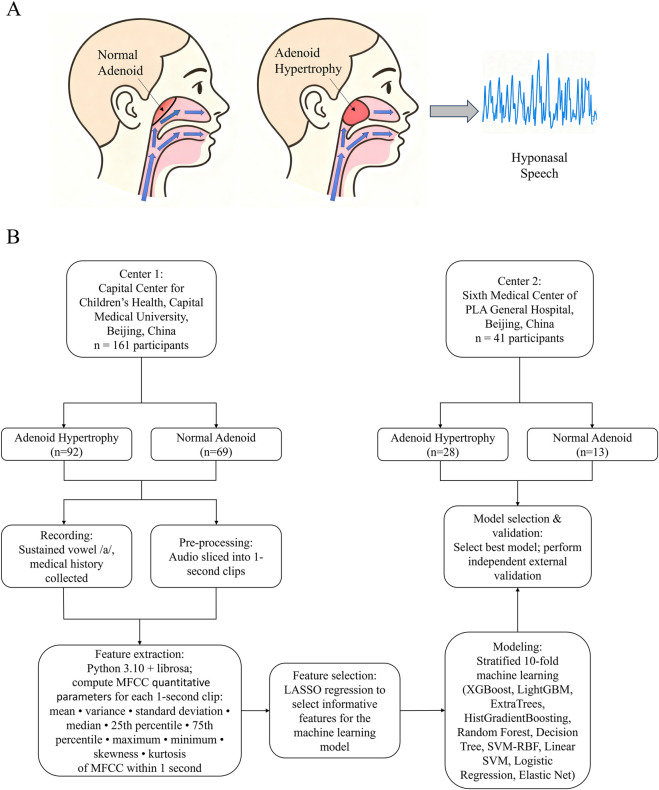
Study overview and MFCC-based acoustic pipeline. **(A)** Adenoid hypertrophy narrows the nasopharyngeal airway and reduces nasal resonance, producing hyponasal speech—the physiological rationale for AH detection from voice. **(B)** Workflow: 202 children were enrolled across two centers (Center 1: AH = 92, controls = 69; Center 2: AH = 28, controls = 13). Sustained/a/was recorded in a quiet room with ambient background noise maintained below 45 dB(A) (monitored at the microphone position) (Sony PCM-D10, 44.1 kHz, 16-bit; microphone ∼10–15 cm in front of the lips at ∼45°). After removing unstable head/tail segments, a 1-s stable clip was extracted. For each child, 13-dimensional MFCCs were computed and summarized into 10 statistics (mean, variance, SD, 25th/75th percentile, min, max, median, skewness, kurtosis) to build a patient-level feature vector, then concatenated with clinical variables. On the development cohort (Center 1), LASSO was used for feature selection; stratified 10-fold CV compared multiple classifiers and the best model was chosen by pooled AUC/AP. A single probability cutoff was fixed by maximizing the Youden index internally and then applied unchanged to the external cohort (Center 2) for independent validation, yielding ROC/PR curves, confusion matrix, calibration, and decision-curve analyses. Abbreviations: AH, adenoid hypertrophy; MFCC, Mel-frequency cepstral coefficients; P25/P75, 25th/75th percentile; AP, average precision; AUC, area under the curve.

Voice signals capture a composite phenotype of upper-airway aerodynamics and vocal-tract resonance. Children with AH frequently present with hyponasality, implying that acoustic features may encode information indicative of upper-airway obstruction (Abdel-Aziz et al., 2023) (Figure 1A). Mel-frequency cepstral coefficients (MFCCs)— descriptors of the short-time spectral envelope—have been widely validated across voice pathology and medical acoustics; compared with end-to-end black-box representations, MFCC-based statistics offer interpretability and cross-device transferability (Tracey et al., 2023; Xie et al., 2023; Jiang et al., 2025; Li et al., 2025)). Meanwhile, routine clinical history-taking yields a set of binary or continuous variables tightly linked to upper-airway pathology (e.g., mouth breathing, snoring, allergic rhinitis, laryngopharyngeal reflux, palatine tonsillar hypertrophy, and basic demographics) without extra resource requirements—signals that may complement acoustic phenotypes (Huang et al., 2018; Marazzato et al., 2020; Tan et al., 2020; Xiao et al., 2022; Zhang et al., 2024).

Against this backdrop, we propose a noninvasive, low-cost, deployable screening strategy for AH that fuses quantitative MFCC statistics with readily obtainable clinical variables, and we evaluate its robustness and translational potential via strict internal validation and independent external validation. To avoid patient-level leakage and overfitting, we aggregate data at the patient level and use stratified 10-fold cross-validation; we select the final model by jointly considering AUC and average precision (AP), and determine a single operating threshold by maximizing the Youden index on the development set, then lock this threshold for direct application to the external cohort—approximating the real-world “train–deploy–apply” pathway (Fluss et al., 2005; Saito and Rehmsmeier, 2015; Rouzrokh et al., 2022). Beyond discrimination (AUC, AP), we report calibration (Brier score, calibration slope and intercept) and decision-curve analysis (DCA) to quantify clinical net benefit across thresholds (Vickers and Elkin, 2006; Van Calster et al., 2019; Huang et al., 2020). We also employ SHAP to characterize the direction and importance of acoustic and clinical predictors, enhancing model interpretability and communication (Lundberg and Lee, 2017).

We enrolled 202 children from two centers in Beijing (Center 1: 92 AH, 69 controls; Center 2: 28 AH, 13 controls). In the development cohort, we extracted 1-s stable segments of sustained/a/, computed 10 subject-level statistics for MFCCs (mean, variance, standard deviation, 25th and 75th percentiles, extrema, skewness, and kurtosis), and integrated them with demographic and upper-airway-related clinical variables. LASSO was used for feature shrinkage/selection, followed by comparison of common classifiers and a two-stage validation with threshold locking (Riley et al., 2024). We aim to demonstrate that an interpretable “voice + history” machine-learning model can serve as a scalable prescreening strategy where endoscopy and imaging are unavailable, reducing unnecessary invasive/radiographic testing without compromising safety and improving resource allocation.

In summary, our contributions are: i. a patient-level AH screening framework combining MFCC-based quantitative acoustics with common clinical features; ii. a deployment-oriented validation chain using AUC/AP-based selection and Youden-index threshold locking, reproduced in an independent external cohort; iii. calibration and DCA to bridge model performance to clinical net benefit; and iv. SHAP-based interpretability clarifying the synergistic roles of acoustic and clinical variables, facilitating cross-site reuse and clinical communication. This work provides a feasible, evidence-based pathway for noninvasive AH screening in primary-care and large-scale settings.

## 2 Methods

### 2.1 Study design and data sources

This dual-center cross-sectional study enrolled 202 children with suspected adenoid hypertrophy (AH) from two pediatric/otolaryngology units in Beijing, China: the Capital Center for Children’s Health, Capital Medical University (Center 1; n = 161) and College of Otolaryngology Head and Neck Surgery, The 6th Medical Center, National Clinical Research Center for Otolaryngologic Diseases, Chinese PLA General Hospital, Beijing, China (Center 2; n = 41). Center 1 contributed 92 AH cases and 69 controls, and Center 2 contributed 28 AH cases and 13 controls, yielding a total of 120 AH and 82 non-AH subjects.

Inclusion criteria were age 3–14 years; clinically suspected AH; ability to cooperate with the standardized voice-recording procedure. Exclusion criteria were previous adenoidectomy or palatine tonsillectomy; significant hearing or speech impairment; history of neck/facial trauma or surgery; congenital craniofacial or airway anomalies (e.g., cleft palate, structural laryngeal lesions) that could affect phonation. On the recording day we collected demographics (age, sex, height, weight) and upper-airway–related histories/symptoms (allergic rhinitis, sinusitis, otitis media, asthma, palatine tonsillar hypertrophy, laryngopharyngeal reflux, recent upper respiratory infection, mouth breathing, snoring, voice disorders, etc.). The overall workflow is shown in Figure 1B.

### 2.2 Reference standard and grouping

Grouping in this study was based on one of two routine first-line diagnostic modalities, nasopharyngoscopy or lateral nasopharyngeal radiography. Both examinations and all image/endoscopic readings were performed by senior otolaryngologists with more than 10 years of relevant clinical experience, in order to ensure inter-center consistency.

For nasopharyngoscopy, adenoid hypertrophy was diagnosed when adenoidal tissue occupied more than 50% of the posterior choanal space; otherwise the child was classified as non-AH. For lateral nasopharyngeal X-ray, children with the adenoid-to-nasopharyngeal (A/N) ratio exceeding 0.7 were assigned to the adenoid hypertrophy group, while those below were classified as the control group. The choice between nasopharyngoscopy and lateral radiography was made by the otolaryngologist according to the child’s age and ability to cooperate. AH labels were derived from routine first-line clinical assessment: flexible nasopharyngoscopy when tolerated, or lateral nasopharyngeal radiography when endoscopy could not be performed (Baldassari and Choi, 2014). Because two accepted modalities were used, some reference-standard variability is possible. The endoscopy-versus-radiography modality was not captured for all participants, so we could not reliably report center-wise modality proportions or conduct modality-stratified sensitivity analyses in the current study.

### 2.3 Audio acquisition and preprocessing

The quantified MFCC-statistics framework adopted in this study—including vowel selection, recording procedure, and environmental noise control—followed the protocol used to develop this feature set (Li et al., 2025). Recordings were performed in an examination room with ambient background noise maintained below 45 dB(A) (monitored at the microphone position). Each child sat upright in a natural, comfortable posture with both feet flat and was instructed to sustain the vowel/a/(“ah”) at a comfortable pitch and loudness. Recordings were monitored on site to ensure >1 s of stable phonation; if < 1 s of stable phonation was obtained or if obvious noise/artifacts were present, the recording was immediately repeated. Two recordings were obtained per subject. Audio was captured with a Sony PCM-D10 recorder in stereo mode (44.1 kHz, 16-bit) with the microphone placed ∼10–15 cm in front of the lips at ∼45°. After removing silent and unstable onset/offset portions, a continuous 1-s window was extracted from the central stable voiced portion for MFCC computation. The two clips from the same subject were processed using the same pipeline to ensure comparability.

### 2.4 MFCC feasectionbture extraction and patient-level aggregation

For each 1-s clip, MFCCs were extracted in Python 3.10 using the librosa library. The MFCC quantification process referenced and followed the methodology reported by Li et al. in the Journal of Voice for voice disorders (Li et al., 2025). The MFCC matrix was summarized by computing 10 descriptive statistics: mean, variance, standard deviation, 25th percentile, 75th percentile, minimum, maximum, median, skewness, and kurtosis. The two recordings from the same child were processed separately and then averaged feature-wise to generate a single, more stable patient-level acoustic profile, which i. reduced within-subject randomness, ii. ensured a “one patient = one row” structure for modeling, and iii. prevented unintentional leakage whereby two clips from the same child would otherwise fall into different folds. Demographic variables (age, height, weight kept as continuous and standardized; sex one-hot/dummy-encoded) and upper-airway–related histories/symptoms (e.g., allergic rhinitis, sinusitis, otitis media, asthma, recent URI, laryngopharyngeal reflux, snoring, mouth breathing, voice disorders, palatine tonsillar hypertrophy) were encoded as 0/1 binary variables and concatenated with the MFCC-derived statistics to form the final input feature set.

### 2.5 Preprocessing

All preprocessing steps were performed within each training fold only. Continuous variables were z-score standardized on the training split and the fitted scaler was applied to the corresponding validation split and to the external set; binary variables were kept as 0/1. The dataset used for modeling was complete; if sporadic missing values were present, they were imputed in the training fold (median for continuous, mode for binary) and the same parameters were carried over to the validation and external data to avoid information leakage. Low-variance and duplicated features were removed before model fitting, following the actual training script.

### 2.6 Feature selection

To mitigate overfitting from the mixed acoustic–clinical feature space, we performed feature selection on the development set (Center 1, n = 161) using logistic regression with L1 regularization (LASSO). AH status was the dependent variable and all MFCC-based plus clinical variables were candidate predictors. The regularization parameter λ was chosen by internal cross-validation using the “minimum error + 1-SE” rule; features with non-zero coefficients at the optimal λ were retained as the most informative subset. On this LASSO-selected subset we refitted a multivariable logistic regression to obtain adjusted odds ratios (ORs) and 95% confidence intervals, which were visualized in a forest plot to show the direction and strength of association.

### 2.7 Model development and internal validation

We compared 10 commonly used classifiers on the same patient-level dataset: logistic regression, Elastic Net logistic regression, linear SVM, RBF-kernel SVM, decision tree, random forest, ExtraTrees, histogram-based gradient boosting, XGBoost, and LightGBM. All linear models used class_weight = “balanced” to account for the moderate AH/non-AH imbalance; for tree/boosting models, the corresponding class-weight or scale_pos_weight options were enabled as in the code.

#### 2.7.1 Cross-validation

Model performance was evaluated on Center 1 using stratified 10-fold cross-validation (shuffle = True, fixed random seed) so that each fold preserved approximately the same AH/non-AH ratio as the full development cohort. Because the audio had already been aggregated to the patient level, additional grouping by patient ID was not required. In every fold, all preprocessing steps (imputation, scaling, variance filtering) were fit on the training split only and then applied to the validation split, matching the leakage-control logic in the training script. Out-of-fold (OOF) predicted probabilities from all 10-folds were concatenated (“pooled”) to produce a single set of development-set predictions for each model.

For each model we computed i. the area under the ROC curve (AUC) and ii. the area under the precision–recall curve (average precision, AP) from the pooled OOF predictions. These two threshold-independent metrics were used to rank models and to pick the final classifier (the one with the highest AUC/AP and the lowest fold-to-fold variability). On the pooled ROC curve of the selected model, we maximized the Youden index (sensitivity + specificity − 1) to obtain a single probability cutoff. This cutoff was derived exclusively from pooled OOF predictions on the development set and was not re-tuned on the external cohort; it was locked and carried forward to external testing.

To match the analysis script, pooled OOF predictions were resampled with 2,000 stratified bootstrap iterations to obtain 95% confidence intervals for both AUC and AP; bootstrap preserved the original class counts in every resample, and percentile CIs were reported.

For the final logistic model, we computed SHAP values using a background sample drawn from the development folds and produced a SHAP summary plot and a mean-absolute-SHAP bar plot, thereby linking both acoustic percentiles (MFCC-25%, MFCC-75%, MFCC-Std) and clinical variables (mouth breathing, allergic rhinitis, laryngopharyngeal reflux, tonsillar hypertrophy, age, weight) to individual predictions.

### 2.8 External validation

The finalized model (feature set, coefficients, scaler, and the internal Youden index cutoff) was applied unchanged to the independent external cohort from Center 2 (n = 41). For each child, MFCC-based features and clinical variables were aligned to the training feature order, scaled with the training scaler, and passed to the model to obtain an AH probability. Subjects were then classified as AH-positive or AH-negative using the locked cutoff. We calculated sensitivity, specificity, positive predictive value (PPV), negative predictive value (NPV), F1-score, and the 2 × 2 confusion matrix, and we also computed external AUC and AP; ROC and PR curves for the external cohort were drawn with 2,000-sample bootstrap 95% CIs exactly as in the internal analysis.

### 2.9 Calibration and decision curve analysis

Calibration on the external cohort was assessed with 10-bin calibration plots, the Brier score, and logistic recalibration to obtain slope and intercept (ideal: slope = 1, intercept = 0). To evaluate clinical usefulness, we ran decision curve analysis (DCA) across threshold probabilities 0.05–0.95 (step 0.01) and compared three strategies: i. model-guided referral for nasopharyngoscopy or lateral nasopharyngeal radiography, ii. test-all, and iii. test-none. Net benefit was reported per 100 patients, and 2,000 bootstrap samples were used to draw 95% CIs for the model curve.

### 2.10 Reproducibility

All analyses were conducted in Python 3.10 using pandas for data handling, librosa for MFCC computation, scikit-learn for model training, cross-validation, calibration, and metrics, xgboost and lightgbm for gradient-boosting models, shap for explainability, and matplotlib/seaborn for figure generation, following the training-and-evaluation scripts archived for this study. All random seeds were fixed and nested parallelism was disabled (n_jobs = 1) to ensure fold-to-fold stability. The scripts are available from the corresponding author upon reasonable request.

### 2.11 Statistical analysis

Continuous variables (age, height, weight) were kept as continuous data, tested for normality, and are presented as mean ± SD if normally distributed (compared by independent-samples t-test) or as median (IQR) if non-normally distributed (compared by Mann–Whitney U test). Categorical variables (upper-airway–related histories/symptoms) are shown as n (%) and were compared using χ^2^ or Fisher’s exact test, as appropriate. All tests were two-sided, and a p value < 0.05 was considered statistically significant. For nonparametric correlation tests, Spearman’s correlation was used.

Model training, stratified 10-fold cross-validation, pooled OOF predictions, Youden index cutoff determination, 2,000-iteration stratified bootstrap CIs for AUC and AP, calibration, SHAP analysis, and decision-curve analysis were implemented in Python 3.10 (pandas, scikit-learn, xgboost, lightgbm, shap, matplotlib), exactly as described above. Conventional descriptive and group-comparison statistics were additionally checked in IBM SPSS Statistics version 27.0. All analyses followed the pre-specified Python scripts used for model training and evaluation to ensure reproducibility.

## 3 Results

### 3.1 Baseline characteristics and feature selection

A total of 202 children were analyzed, including 120 with adenoid hypertrophy (AH) and 82 controls. The overall baseline comparison between AH and non-AH groups is presented in Table 1. Compared with controls, children with AH were slightly younger (median 6.21 vs. 7.10 years, P = 0.04) and showed a clearly higher burden in following features: mouth breathing (63.3% vs. 24.4%, P < 0.01), snoring (45.0% vs. 26.8%, P = 0.01), palatine tonsillar hypertrophy (54.2% vs. 32.9%, P < 0.01), allergic-rhinitis history (73.3% vs. 37.8%, P < 0.01), and laryngopharyngeal reflux (50.0% vs. 26.8%, P < 0.01). Height and weight were broadly comparable between groups (both P > 0.1), suggesting that the differences were not simply driven by somatic growth. Among the acoustic descriptors, only the 25th percentile of the MFCCs was significantly lower in the AH group than in controls (p < 0.01), while the other MFCC statistics did not differ significantly (median −27.73 vs. −32.16, Table 1).

**TABLE 1:** Baseline characteristics of participants stratified by adenoid hypertrophy (pooled across both centers).

Variable	Control (n = 82)	Adenoid hypertrophy (n = 120)	t/U/χ^2^	*P* value
Age	7.10 (5.88, 8.80)	6.21 (4.98, 7.79)	5766.00	0.04
Gender	​	​	5.61	0.02
*girl*	44 (53.66%)	43 (35.83%)	​	​
*boy*	38 (46.34%)	77 (64.17%)	​	​
Height	125.00 (115.25, 135.75)	121.50 (110.75, 130.00)	5462.50	0.18
Weight	23.00 (20.00, 30.00)	23.25 (19.45, 31.25)	4865.00	0.89
Mouth breathing	​	​	28.08	<0.01
*negative*	62 (75.61%)	44 (36.67%)	​	​
*positive*	20 (24.39%)	76 (63.33%)	​	​
Snoring	​	​	6.10	0.01
*negative*	60 (73.17%)	66 (55.00%)	​	​
*positive*	22 (26.83%)	54 (45.00%)	​	​
Tonsil hypertrophy	​	​	8.03	<0.01
*negative*	55 (67.07%)	55 (45.83%)	​	​
*positive*	27 (32.93%)	65 (54.17%)	​	​
AR history	​	​	23.96	<0.01
*negative*	51 (62.20%)	32 (26.67%)	​	​
*positive*	31 (37.80%)	88 (73.33%)	​	​
Sinusitis history	​	​	0.99	0.32
*negative*	75 (91.46%)	103 (85.83%)	​	​
*positive*	7 (8.54%)	17 (14.17%)	​	​
Otitis media history	​	​	1.07	0.30
*negative*	74 (90.24%)	101 (84.17%)	​	​
*positive*	8 (9.76%)	19 (15.83%)	​	​
Asthma	​	​	NA	0.45
*negative*	78 (95.12%)	117 (97.50%)	​	​
*positive*	4 (4.88%)	3 (2.50%)	​	​
Respiratory infection in 2 weeks	​	​	0.58	0.45
*negative*	68 (82.93%)	93 (77.50%)	​	​
*positive*	14 (17.07%)	27 (22.50%)	​	​
Laryngopharyngeal reflux	​	​	9.91	<0.01
*negative*	60 (73.17%)	60 (50.00%)	​	​
*positive*	22 (26.83%)	60 (50.00%)	​	​
Voice disorder	​	​	NA	0.40
*negative*	79 (96.34%)	118 (98.33%)	​	​
*positive*	3 (3.66%)	2 (1.67%)	​	​
MFCC-mean	−28.08 (−33.72, −25.92)	−30.07 (−33.29, −27.19)	5464.00	0.18
MFCC-var	14981.10 (11576.33, 17528.80)	13600.19 (11622.58, 16806.54)	5495.00	0.16
MFCC-std	122.36 (107.58, 132.39)	116.17 (107.25, 129.64)	5506.00	0.15
MFCC-25%	−27.73 ± 7.64	−32.16 ± 8.09	3.91	<0.01
MFCC-75%	11.28 (9.07, 15.78)	11.88 (8.93, 15.30)	4794.00	0.76
MFCC-min	−375.05 (−426.69, −331.08)	−371.75 (−407.14, −330.09)	4634.00	0.48
MFCC-max	172.64 (162.26, 183.75)	174.07 (161.00, 184.48)	4925.00	0.99
MFCC-median	−8.34 ± 4.87	−8.74 ± 5.28	0.54	0.59
MFCC-skew	−1.59 (−1.88, −1.27)	−1.56 (−1.73, −1.38)	4763.00	0.70
MFCC-kurtosis	2.66 (2.15, 3.28)	2.61 (2.18, 2.93)	5198.00	0.50

A two-sided P < 0.05 was considered statistically significant.

Continuous variables are presented as mean ± standard deviation (Mean ± SD) if normally distributed and compared using the independent-samples t-test; otherwise, they are shown as median (interquartile range) and compared using the Mann–Whitney U test.

Categorical variables are expressed as counts (percentages) and compared using Pearson’s chi-square test. For 2 × 2 tables with expected counts <5, Fisher’s exact test was applied. For Fisher’s exact test, the “t/U/χ^2^” column is marked as NA, and only the P value is reported.

Because the cohort came from two hospitals, we further split the baseline by center: Supplementary Table S1 summarizes healthy controls from Center 1 vs. Center 2, and Supplementary Table S2 summarizes AH patients from the two centers. Overall symptom patterns were similar across centers, but AH cases from Center 2 tended to be older and to have a slightly higher proportion of concomitant airway symptoms, which provided a realistic distribution shift for external validation. Therefore, all subsequent model training, cutoff determination, and pooled internal evaluation were performed on Center 1 only, and Center 2 was kept as an untouched external test set.

**FIGURE 2:**
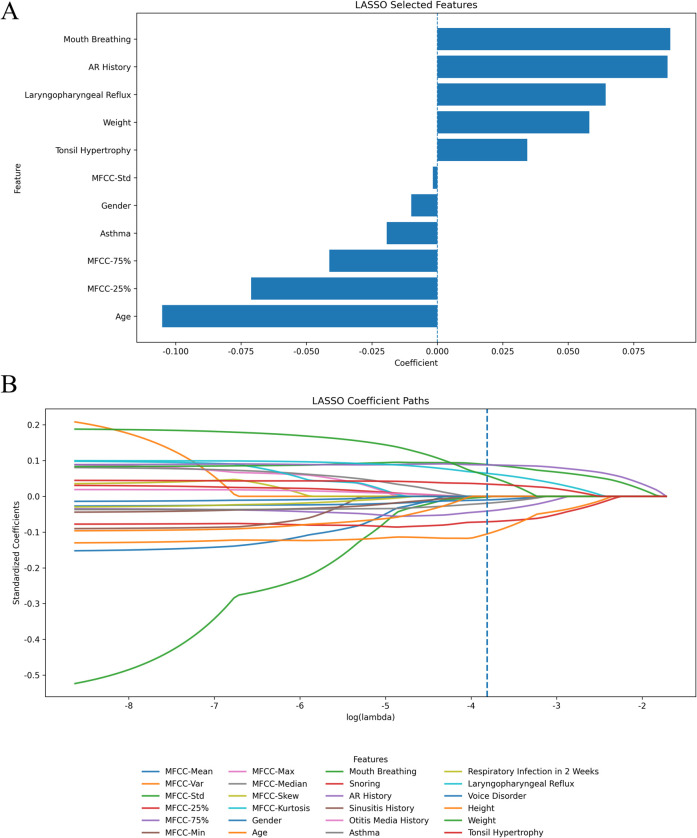
LASSO-based feature selection on the development cohort. **(A)** Non - zero coefficients at the optimal penalty (1-SE rule) after z-score standardization for continuous variables and 0/1 encoding for binaries. Bar length indicates effect size; sign indicates direction of association with adenoid hypertrophy (positive = higher risk, negative = lower risk). **(B)** Regularization paths (standardized coefficients vs. log(λ)); the dashed line marks the selected λ. Features whose paths shrink to zero at this λ were excluded from the final set. Abbreviations: MFCC-25%/75% = 25th/75th percentile of MFCC values across frames; MFCC-Std = standard deviation; AR = allergic rhinitis; LPR = laryngopharyngeal reflux.

**FIGURE 3:**
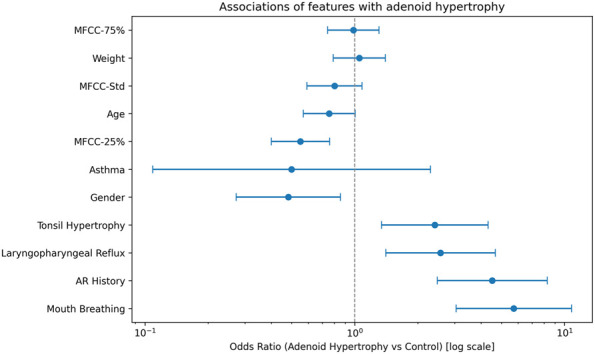
Adjusted associations between LASSO-selected predictors and adenoid hypertrophy. Multivariable logistic regression was fitted on the development cohort using only the variables retained by LASSO (MFCC-25%, MFCC-75%, MFCC-Std, age, weight, tonsil hypertrophy, mouth breathing, allergic rhinitis, laryngopharyngeal reflux [LPR], gender, and asthma). Continuous variables were standardized; odds ratios (dots) therefore represent the change in odds of adenoid hypertrophy per 1-SD increase in the predictor, while binary variables are shown as 1 vs. 0. Horizontal lines indicate 95% confidence intervals, and the vertical dashed line marks OR = 1 (no effect).

On this development cohort we then applied L1-penalized logistic regression (LASSO) to the pooled acoustic-plus-clinical feature space to reduce dimensionality and avoid overfitting (Figure 2). At the penalty selected by the 1-SE rule, the model retained a mixed set of predictors: MFCC-25%, MFCC-75%, MFCC-Std, age, weight, tonsil hypertrophy, mouth breathing, allergic rhinitis, laryngopharyngeal reflux (LPR), gender, and asthma, indicating that both acoustic dispersion features and classical upper-airway symptoms contributed signal. These LASSO-selected variables were entered into a multivariable logistic regression to obtain adjusted odds ratios and 95% CIs, which were displayed in a forest plot (Figure 3). After standardization, higher MFCC-75% and MFCC-Std, together with mouth breathing, allergic rhinitis, LPR and tonsil hypertrophy, were associated with increased odds of AH, while younger age and lower MFCC-25% were also linked to AH. This integrated presentation keeps the descriptive cohort profile together with the data-driven selection step, and is concise enough for the main text.

To evaluate whether the selected MFCC distribution features primarily reflect age-related maturation within the broad developmental range, we assessed monotonic associations between age and the key MFCC features in the development cohort (Center 1, n = 161) using Spearman’s rank correlation. Correlations were small and non-significant: MFCC-Std (ρ = 0.09, 95% CI −0.07 to 0.25, P = 0.26), MFCC-25% (ρ = −0.09, 95% CI −0.25 to 0.07, P = 0.25), and MFCC-75% (ρ = 0.08, 95% CI −0.08 to 0.23, P = 0.33). These results suggested that the selected MFCC predictors were not associated with ages.

### 3.2 Internal validation

**FIGURE 4:**
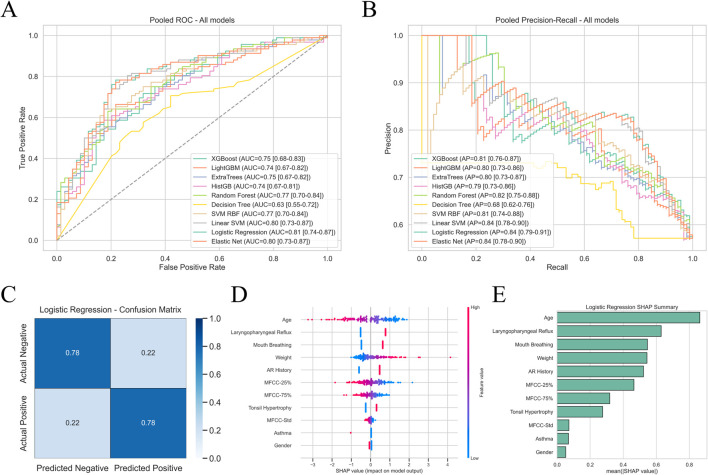
Internal 10-fold development results (Center 1, n = 161). **(A)** Pooled ROC curves of 10 candidate classifiers on Center 1; logistic regression showed the best discrimination (AUC 0.81, 95% CI 0.74–0.87). **(B)** Pooled precision–recall curves of the same models; logistic regression also achieved the highest AP (0.84, 95% CI 0.79–0.91). **(C)** Confusion matrix of the final logistic model at the internally selected Youden cutoff (0.56), with sensitivity = 0.78 and specificity = 0.78. **(D)** SHAP beeswarm plot showing that age, laryngopharyngeal reflux, mouth breathing, weight, allergic rhinitis history and MFCC percentiles contributed most. **(E)** SHAP global importance ranking confirming complementary value of MFCC statistics and clinical variables.

On the patient-level development cohort (Center 1), we benchmarked ten commonly used classifiers with stratified 10-fold cross-validation (Figures 4A,B). Overall, regularized linear models clearly outperformed tree/boosting models. Logistic regression achieved the best discrimination, with AUC 0.81 (95% CI 0.74–0.87) and AP 0.84 (95% CI 0.79–0.91). Elastic Net and linear SVM showed almost overlapping curves (AUC = 0.80; AP = 0.84), indicating that, for this sample size and feature space, a regularized linear decision boundary is sufficient to separate AH from non-AH. In contrast, non-linear/tree-based models (random forest, HistGB, ExtraTrees, XGBoost, LightGBM) clustered in the AUC 0.74–0.77 and AP 0.79–0.82 range, and the single decision tree was the weakest (AUC 0.63; AP 0.66), matching the ranking shown in Figure 4.

Following the actual training script, we first ranked models by the pooled out-of-fold AUC and AP, and then selected logistic regression as the final model. On the pooled ROC curve of this model, we maximized Youden’s J to obtain a single operating cutoff of 0.56 and explicitly locked it so that the threshold would not be re-tuned on the external cohort, thereby preventing information leakage. At this cutoff, the confusion matrix showed sensitivity 0.78 and specificity 0.78 (Figure 4C), indicating a balanced tradeoff between missing AH cases and over-calling normal children. Details of the model-specific performance are provided in Table 2.

**TABLE 2:** Pooled cross-validated performance of candidate classifiers on the development cohort.

Model	Accuracy	Sensitivity	Specificity	Youden index	Micro F1	Macro F1	Matthews coefficient
XGBoost	0.70	0.72	0.68	0.40	0.70	0.70	0.40
LightGBM	0.71	0.66	0.78	0.45	0.71	0.71	0.44
ExtraTrees	0.70	0.75	0.62	0.37	0.70	0.69	0.38
HistGB	0.69	0.58	0.84	0.42	0.69	0.69	0.42
Random forest	0.71	0.63	0.81	0.44	0.71	0.71	0.44
Decision tree	0.65	0.71	0.58	0.29	0.65	0.64	0.29
SVM RBF	0.72	0.64	0.83	0.47	0.72	0.72	0.47
Linear SVM	0.78	0.80	0.74	0.54	0.78	0.77	0.54
Logistic regression	0.78	0.78	0.78	0.57	0.78	0.78	0.56
Elastic net	0.78	0.77	0.78	0.55	0.78	0.77	0.55

To address uncertainty, the pooled out-of-fold predictions were resampled with 2,000 stratified bootstrap iterations to derive 95% CIs for both AUC and AP; these CIs are the ones reported above, so the numerical results are fully consistent with the code.

Explainability analysis (Figures 4D,E) showed that age was the strongest negative contributor, while laryngopharyngeal reflux, mouth breathing, weight, allergic rhinitis history, and tonsillar hypertrophy were prominent positive contributors. Acoustic statistics—particularly MFCC-25% (negative), MFCC-75% (positive), and MFCC-Std (positive)—added complementary signal beyond clinical variables, which supports the premise of combining voice with routine clinical data. Considering the highest AUC/AP, fold-to-fold stability and transparent interpretability, logistic regression was finally selected, and the 0.56 cutoff was carried forward unchanged to external validation.

### 3.3 Independent external validation

**FIGURE 5:**
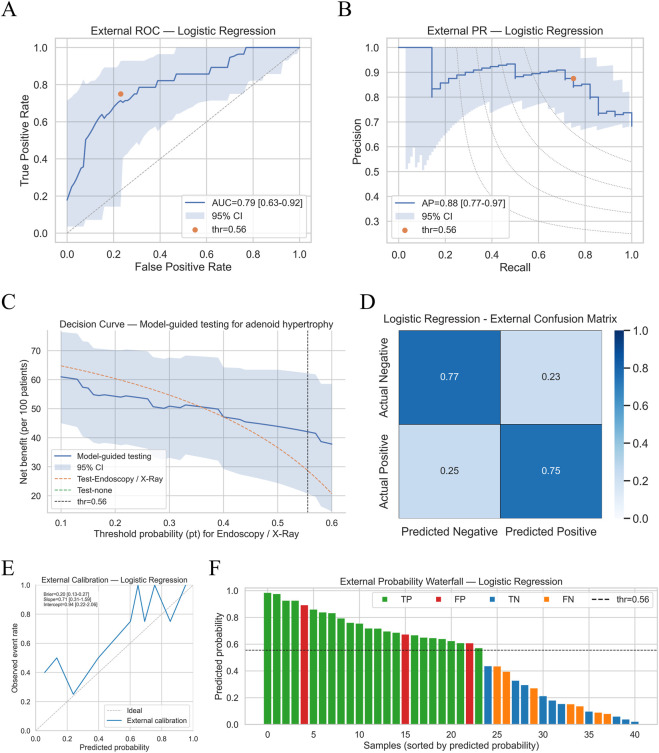
Independent external validation cohort (Center 2, n = 41). **(A)** External ROC of the final logistic model (AUC 0.79, 95% CI 0.63–0.92; orange dot = locked cutoff 0.56). **(B)** External PR curve (AP 0.88, 95% CI 0.78–0.97; orange dot = cutoff 0.56). **(C)** Decision curve showing higher net benefit than test-all/test-none across clinically relevant thresholds. **(D)** Confusion matrix at the locked cutoff (sensitivity 0.75, specificity 0.77, F1 0.76). **(E)** Calibration plot (Brier 0.20; slope 0.71; intercept 0.94). **(F)** Probability waterfall plot sorted by predicted risk (TP/FP/TN/FN labeled; dashed line = 0.56).

In the independent external cohort (Center 2, n = 41), the locked logistic regression model largely maintained the discriminative ability observed in development. The ROC curve yielded an AUC of 0.79 (95% CI 0.63–0.92) and the precision–recall curve an AP of 0.88 (95% CI 0.78–0.97), supporting between-center transportability of the model. Given the limited external sample size, confidence intervals are necessarily wider, and larger external cohorts are warranted to improve the precision of performance estimates (Figures 5A,B). Using the probability cutoff of 0.56 that had been fixed *a priori* from stratified 10-fold CV, the model achieved sensitivity 0.75, specificity 0.77, and an F1-score of 0.76, with the 2 × 2 confusion matrix showing a balanced pattern of true positives and true negatives (Figure 5D). Calibration performance was acceptable: the calibration curve followed the ideal line with modest underestimation at the upper end, the Brier score was 0.20, and logistic recalibration yielded a slope of 0.71 and intercept of 0.94 (Figure 5E). Decision curve analysis demonstrated that, across clinically plausible threshold probabilities for deciding on nasopharyngoscopy or lateral nasopharyngeal radiography, the model-guided testing strategy delivered a higher net benefit than either “test-all” or “test-none,” supporting its use as a prescreening/triage tool rather than a stand-alone diagnostic (Figure 5C). The external probability waterfall plot further illustrated patient-level risk separation: most true AH cases clustered above the 0.56 line, while most controls were ranked in the lower-probability zone, with only a small number of false positives/false negatives near the decision boundary (Figure 5F).

## 4 Discussion

We propose and externally test an interpretable, low-barrier, deployment-oriented prescreening model for adenoid hypertrophy (AH) that fuses quantitative MFCC statistics with routine clinical variables within a patient-level, leakage-controlled framework. In a small independent external cohort, the locked-threshold model showed preservation of discrimination with acceptable calibration and net benefit, suggesting potential utility as a pre-endoscopy triage tool. Larger, prospective multicenter validations are needed before broad implementation.

The model demonstrated stable discrimination internally and externally (internal AUC = 0.81, AP = 0.84; external AUC = 0.79, AP = 0.88) and achieved clinically usable sensitivity/specificity and F1 at the locked cutoff. External calibration (Brier = 0.20, slope = 0.71, intercept = 0.94) and decision-curve analysis indicated higher net benefit than the “test-all” or “test-none” strategies across a range of thresholds Compared with auscultation-based approaches that rely on dedicated electronic or wearable stethoscopes—thereby imposing hardware and workflow barriers—our pipeline requires only a 1-s sustained/a/recording, achieving low deployment cost and operational simplicity (Lee et al., 2025; Xiao et al., 2025). It also avoids radiation and invasiveness intrinsic to lateral radiography (A/N ratio) and nasopharyngoscopy, which limit scalability for mass or school screening (Fujioka et al., 1979; Baldassari and Choi, 2014).

Beyond “AI for pediatric sleep/airway” in general (Gutiérrez-Tobal et al., 2022), our contribution is to explicitly couple interpretable acoustic descriptors with point-of-care clinical features, with SHAP supporting transparent attribution and clinical communication. In our data, MFCC percentiles and dispersion (e.g., 25%, 75%, standard deviation) show consistent group differences between AH and non-AH (Xu et al., 2024). From a speech-physiology perspective, increased nasal obstruction alters nasal-oral-pharyngeal coupling, redistributing the spectral envelope via antiresonances; MFCCs, as compact encoders of the short-term spectral envelope, are well suited to capture these changes (Diehl, 2008; Remez et al., 2011; Verma et al., 2023). Clinical further documents nasal resonance changes around adenoid surgery, reinforcing the obstruction-to-acoustics bridge (Abdel-Aziz et al., 2023). Our quantified MFCC statistics compress high-dimensional frame-level acoustic matrices into tabular features that integrate seamlessly with point-of-care clinical variables; looking ahead, this low-cost “quantified acoustics + clinical features” pipeline could generalize to broader disease prescreening and triage, better supporting frontline clinicians (Jiang et al., 2025; Li et al., 2025).

In this research, we used patient-wise aggregation and splits with Stratified 10-fold CV to prevent leakage across multiple clips from the same child and to match deployment to new patients (Bradshaw et al., 2023; Wilimitis and Walsh, 2023). Models were ranked by a joint AUC–AP criterion to respect class imbalance (Saito and Rehmsmeier, 2015), and a single Youden-derived cutoff was locked on the development cohort and applied without re-tuning externally—mirroring go-live practice and reducing optimism bias (Youden, 1950). Decision curve analysis showed higher net benefit over “test-all/none” across a clinically relevant threshold range (Vickers and Elkin, 2006), and external calibration (Brier/slope/intercept) supported acceptable agreement between predicted and observed risks (Van Calster et al., 2019). Overall, the approach strikes a pragmatic balance between non-invasiveness, low barriers, interpretability, and deployment readiness, serving as a front-end prescreener before endoscopy/imaging.

The 3–14-year age range spans substantial maturation of the pediatric phonatory and vocal-tract system, which could confound acoustic comparisons. Importantly, age was retained by LASSO feature selection and intentionally was included as a predictor in the final multivariable model based on its established clinical and maturational relevance, rather than because age is unrelated to voice features. As a complementary check, we examined monotonic associations between age and the key MFCC distribution features in the overall cohort (n = 202) using Spearman’s rank correlation and observed weak, non-significant relationships: MFCC-Std (ρ = 0.08, 95% CI −0.06 to 0.22, P = 0.23), MFCC-25% (ρ = −0.08, 95% CI −0.22 to 0.07, P = 0.27), and MFCC-75% (ρ = 0.08, 95% CI −0.06 to 0.22, P = 0.25). Analyses repeated separately in each center yielded consistent patterns, suggesting that these MFCC predictors are unlikely to be simple proxies for age-related maturation. Together with explicit age adjustment in multivariable modeling, this helps mitigate concerns about developmental confounding.

This study has several constraints. First, the sample is modest and drawn from two Beijing centers, so generalizability across regions, languages/dialects, and recording environments remains uncertain. Second, we relied on hand-crafted, statistics-based voice features rather than end-to-end neural networks; while this improves interpretability and reduces data demands, it may underuse high-dimensional structure exploitable by deep models. Third, external validation was single-center (n = 41) and retrospective rather than workflow-embedded, limiting inferences about operational effectiveness and calibration drift. Because AH status was determined using either endoscopy or lateral radiography in routine practice—a pragmatic approach that aligns with best-practice recommendations to use endoscopy when tolerated and radiography when cooperation is limited (Baldassari and Choi, 2014)—some degree of reference-standard variability is possible. Moreover, the endoscopy-versus-radiography modality was not captured for all participants, precluding center-wise reporting of modality distribution and modality-stratified sensitivity analyses. Such heterogeneity may contribute to non-differential label noise and potentially attenuate observed associations and performance estimates, implying that our results may be conservative. Future prospective studies will standardize the reference assessment and/or explicitly record the diagnostic modality to enable center-wise reporting and modality-stratified evaluation. Finally, we did not systematically quantify device variability (smartphones vs. dedicated recorders vs. clinic hardware) or task diversity (multiple vowels, connected speech, counting), both of which could affect point-of-care robustness.

We will build a prospective, multi-center cohort spanning diverse linguistic communities and acoustic settings; preregister protocols; harmonize recording scripts, microphones, and metadata; and institute continuous quality control. We will benchmark modern representation-learning approaches (self-supervised audio encoders and lightweight end-to-end architectures) against our interpretable pipeline under strict data-governance and privacy safeguards. To support deployment, we will quantify device effects, develop calibration-transfer and model-updating procedures, and design task-agnostic feature fusion covering multiple sustained vowels and connected speech. We also plan pragmatic, workflow-embedded evaluations comparing model-assisted triage with usual care, integrate outputs with endoscopy/imaging pathways, and conduct subgroup fairness, error-analysis, and cost-effectiveness studies to guide threshold selection and scale-up.

## 5 Conclusion

This study presents an interpretable, low-burden prescreening model for pediatric adenoid hypertrophy that integrates quantified MFCC statistics from sustained/a/with routine clinical variables under a patient-level framework. The locked-threshold logistic regression model achieved good discrimination in development and preserved performance in an independent external center, suggesting a certain degree of between-center transportability in a real-world setting. Given the modest external sample size and reference-standard heterogeneity, further prospective multicenter validation with standardized assessment and recording protocols is warranted before broad implementation.

## Data Availability

The original contributions presented in the study are included in the article/Supplementary Material, further inquiries can be directed to the corresponding authors.
